# Radiologic-Pathologic Correlation in Biliary Cystic Neoplasms: Insights From a Single-Centre Experience

**DOI:** 10.7759/cureus.104277

**Published:** 2026-02-26

**Authors:** Evangelia Florou, Rakshana Munusamy, Memoona Mukhtar, Nuran Seneviratne, Yoh Zen, Parthi Srinivasan, Andreas Prachalias

**Affiliations:** 1 Hepato-Pancreato-Biliary Surgery, King’s College Hospital, London, GBR; 2 Interventional Radiology, King’s College Hospital, London, GBR; 3 Pathology and Laboratory Medicine, King’s College Hospital, London, GBR; 4 Hepato-Pancreato-Biliary Surgery and Liver Transplantation, London Bridge Hospital, London, GBR

**Keywords:** biliary cystadenocarcinoma, biliary cystadenoma, biliary cystic neoplasm, hepatic cystic lesion, mri, radiologic–pathologic correlation

## Abstract

Background

Biliary cystic neoplasms (BCNs), comprising biliary cystadenomas (BCAs) and cystadenocarcinomas (BCACs), are rare cyst-forming epithelial tumours of the liver and biliary tract. Differentiation between benign and malignant lesions remains challenging preoperatively, and current imaging criteria lack sufficient discriminatory power. This study aimed to correlate preoperative imaging features with final histology to better identify radiologic markers of malignancy in BCN.

Methodology

A retrospective analysis was conducted on 42 histologically confirmed cases of BCAs or BCACs discussed at a specialist hepatopancreatobiliary multidisciplinary team between 2010 and 2023. All cases had preoperative cross-sectional imaging (CT and/or MRI or magnetic resonance cholangiopancreatography). Imaging studies were reviewed for features including wall enhancement, septations, mural nodules, calcification, and internal fluid complexity. Histology was categorised into low-grade dysplasia (LGD), high-grade dysplasia (HGD), or invasive carcinoma (BCAC).

Results

Of the 42 cases, 39 (93%) were LGD BCA, two (5%) were HGD BCA, and one (2%) was BCAC. Wall enhancement was significantly more common in advanced histology (67%) compared to LGD (28%), and thick fluid density also correlated with advanced pathology (33% vs. 8%). Mural nodules were present only in LGD cases (10%) and absent in HGD or BCAC. The sensitivity and specificity of wall enhancement for advanced histology were 67% and 72%, respectively. Notably, ovarian-type stroma (OTS), a diagnostic criterion in the WHO 2022 classification, was absent in 24% of cases, yet these were still reported as BCAs based on traditional morphological features.

Conclusions

Wall enhancement and complex fluid characteristics are the most suggestive imaging features of advanced BCN; however, significant overlap with benign lesions limits their diagnostic accuracy. Mural nodules were not predictive of malignancy in this cohort. Our findings support surgical resection of all complex or atypical cystic liver lesions and highlight the variability in real-world histopathological classification, especially regarding OTS. Multidisciplinary consensus and histologic evaluation remain critical for definitive diagnosis.

## Introduction

Biliary cystic neoplasms (BCNs) include biliary cystadenomas (BCAs) and cystadenocarcinomas (BCACs) [1]. BCNs account for fewer than 5% of all cystic liver lesions and exhibit a profound female predominance of approximately about 90% [1,2]. The vast majority arise within the intrahepatic biliary tree, and the most widely accepted theory supports malignant transformation of normal biliary epithelium through progressive dysplastic changes [3].

BCA with low-grade dysplasia (LGD) may progress to high-grade dysplasia (HGD), which, in turn, may evolve into invasive BCAC [1,2]. The presence of ovarian-type stroma (OTS) is a cardinal histological feature of mucinous cystic neoplasms (MCNs) under the World Health Organization (WHO) criteria, and is commonly seen in BCA [2,4,5]. Symptoms are often nonspecific and related to mass effect, such as abdominal discomfort due to compression of adjacent structures [1]. Extrahepatic lesions may present with obstructive jaundice [1].

When evaluating a complex hepatic cystic lesion, the differential diagnosis includes simple hepatic cyst, BCN, liver abscess, polycystic liver disease, cystic degeneration of metastases, and other rare primary biliary lesions such as lymphangioma, mesenchymal hamartoma, cystic haemangioma, and teratoma [1,4]. Because BCNs possess malignant potential, and preoperative imaging lacks pathognomonic features, surgical resection of all complex cystic liver lesions is generally recommended following multidisciplinary team (MDT) discussion [1,4].

This study aims to correlate preoperative imaging features with final histological findings in resected BCNs at a tertiary hepato-pancreato-biliary (HPB) centre and contribute to a better understanding of this rare entity.

## Materials and methods

This retrospective, single-centre study was conducted using anonymised data obtained from routine clinical records and imaging archives. No patient contact or intervention occurred, and no identifiable information was used. In accordance with institutional policy and UK national guidelines, formal Research Ethics Committee approval and individual patient consent were not required.

This retrospective, single-centre study was conducted at a tertiary HPB unit. A total of 42 histologically confirmed cases of BCA or BCAC were identified among 230 cystic liver lesions discussed at the MDT meeting between 2010 and 2023.

Preoperative imaging included CT in 88% of patients and/or MRI with magnetic resonance cholangiopancreatography (MRCP) in 64%. Both modalities were available in 52% of cases. All available imaging studies were reviewed for the presence of wall enhancement, internal septations, mural nodules, calcification, and non-simple (thick) fluid density. MRI-specific parameters included T1/T2 signal intensity, diffusion restriction, and biliary tree communication.

Final histopathological diagnoses were categorised as LGD BCA, HGD BCA, or BCAC, based on the degree of epithelial atypia and evidence of invasion.

## Results

Of the 230 MDT discussions concerning cystic liver lesions, 42 patients with histologically confirmed BCN were included after excluding cases with clearly benign diagnoses, alternative pathologies, or incomplete clinical or imaging data. Surgical resection was undertaken in all cases based on consensus MDT decision, prompted by the presence of complex features on cross-sectional imaging.

The mean age at diagnosis was 48.5 years (range = 22-77 years), with a marked female predominance (98%). Most patients (88.1%) were symptomatic at presentation, while 11.9% were diagnosed incidentally. Preoperative CT was available in 88.1% of cases, MRI/MRCP in 64.3%, and both modalities were available in 52.4%. No patients underwent PET imaging.

Radiologic features included enhancing septa in 45.2% of patients, wall enhancement in 31.0%, multiloculated morphology in 64.3%, calcification in 19.0%, mural nodules in 9.5%, and non-simple (thick) fluid density in 9.5%. The mean cyst size was 110.4 mm (range = 30-260 mm). Most lesions were centrally or bi-lobarly located (59.5%), with right and left lobe involvement in 19.0% each, and a single case (2.4%) located in the extrahepatic biliary tree.

Histopathologic examination confirmed LGD in 39 (93%) patients, HGD in two (4.8%) patients, and invasive BCAC in one (2.4%) patient. Advanced histology (HGD or BCAC) was thus present in three (7.1%) cases. OTS was identified in 76.2% of specimens and absent in 23.8%.

When stratified by histological grade, wall enhancement demonstrated the strongest correlation with advanced disease (sensitivity = 67%, specificity = 72%, odds ratio = ~5.1). Although rare, thick fluid content had high specificity (92%) and an odds ratio of approximately 6.0. Enhancing septa were observed frequently but showed limited discriminatory value (specificity = 54%). Notably, mural nodules were not predictive of advanced histology in this cohort, as they were observed exclusively in LGD lesions.

A detailed summary of the diagnostic performance of radiologic features is presented in Table 1. Wall enhancement was assessed using contrast-enhanced cross-sectional imaging, including both CT and MRI, as available for each case.

**Table 1:** Diagnostic accuracy of radiological features in predicting advanced histology. The table presents the diagnostic performance of selected radiological features in differentiating advanced histology (HGD or BCAC) from LGD BCA. Sensitivity, specificity, and OR were calculated using the final histopathology. HGD: high-grade dysplasia; BCAC: biliary cystadenocarcinoma; LGD: low-grade dysplasia; BCA: biliay cystadenoma; OR: odds ratio

Feature	Advanced histology (n = 3)	LGD histology (n = 39)	Sensitivity	Specificity	OR	Interpretation
Wall enhancement	2	11	66.7%	71.8%	5.1	Strongest correlating feature
Enhancing septa	1	18	33.3%	53.8%	0.6	Weak discriminatory value
Thick fluid density	1	3	33.3%	92.3%	6.0	Rare but highly specific
Calcification	1	7	33.3%	82.1%	2.3	Modest correlation
Mural nodules	0	4	0.0%	89.7%	0.0	Not predictive in this cohort

**Figure 1:**
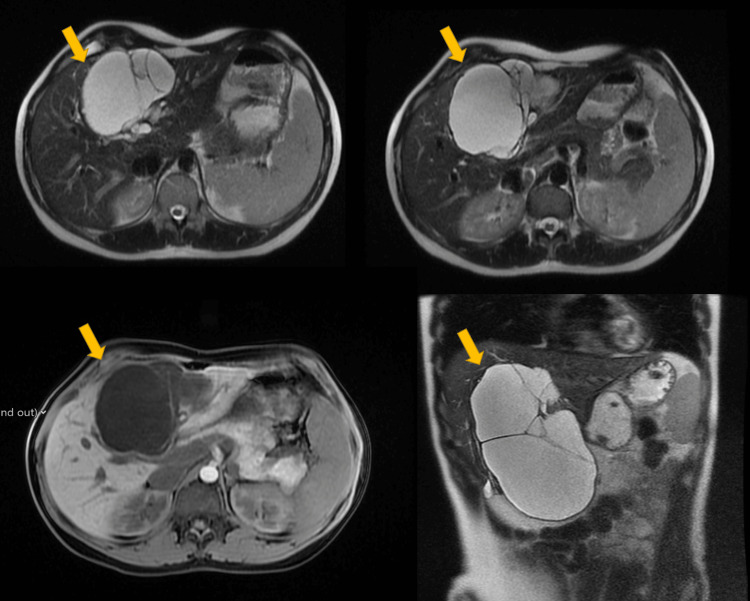
Axial and coronal MRI appearances of LGD BCA. T2-weighted axial (top row) and coronal (bottom right) MRI images demonstrate a well-defined, multilocular cystic lesion (yellow arrows) in the liver, with thin internal septations and high T2 signal intensity. The axial T1-weighted image (bottom left) shows low signal intensity without enhancement or mural nodularity. These features are characteristic of LGD BCA, with no evidence of invasive behaviour or complex internal architecture. LGD: low-grade dysplasia; BCA: biliary cystadenoma

**Figure 2:**
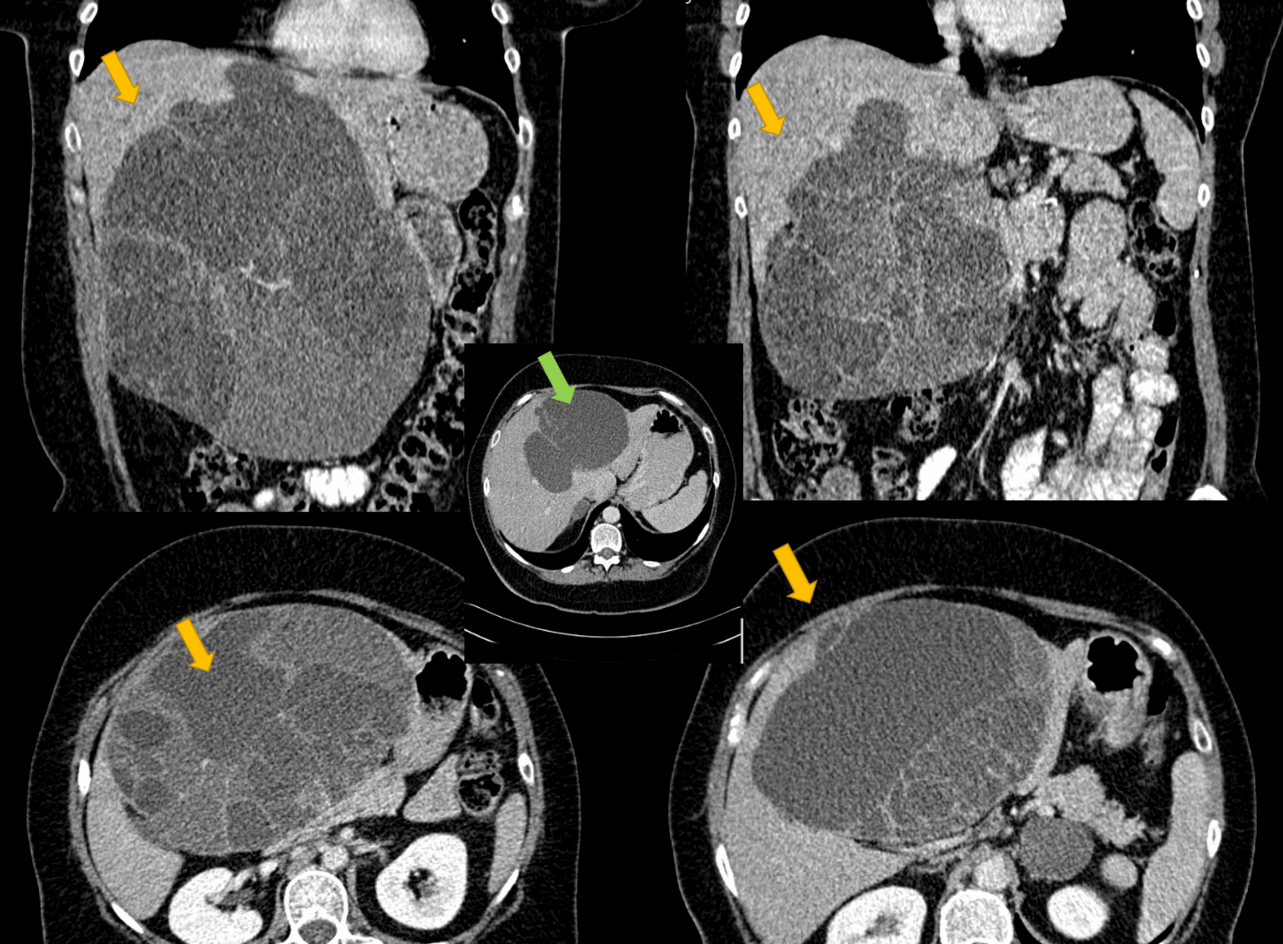
Multiphase CT imaging of HGD BCA. Axial and coronal contrast-enhanced CT images demonstrate a large multiloculated cystic hepatic lesion (yellow arrows) with thick enhancing septations and irregular wall enhancement. Internal fluid content is heterogeneous, suggesting mucinous or proteinaceous material. These features are associated with advanced histology and prompted surgical resection. The central image (green arrow) shows the same lesion on non-contrast CT, illustrating the absence of pre-existing hyperdensity, and highlighting the enhancement pattern on post-contrast images. HGD: high-grade dysplasia; BCA: biliary cystadenoma

**Figure 3:**
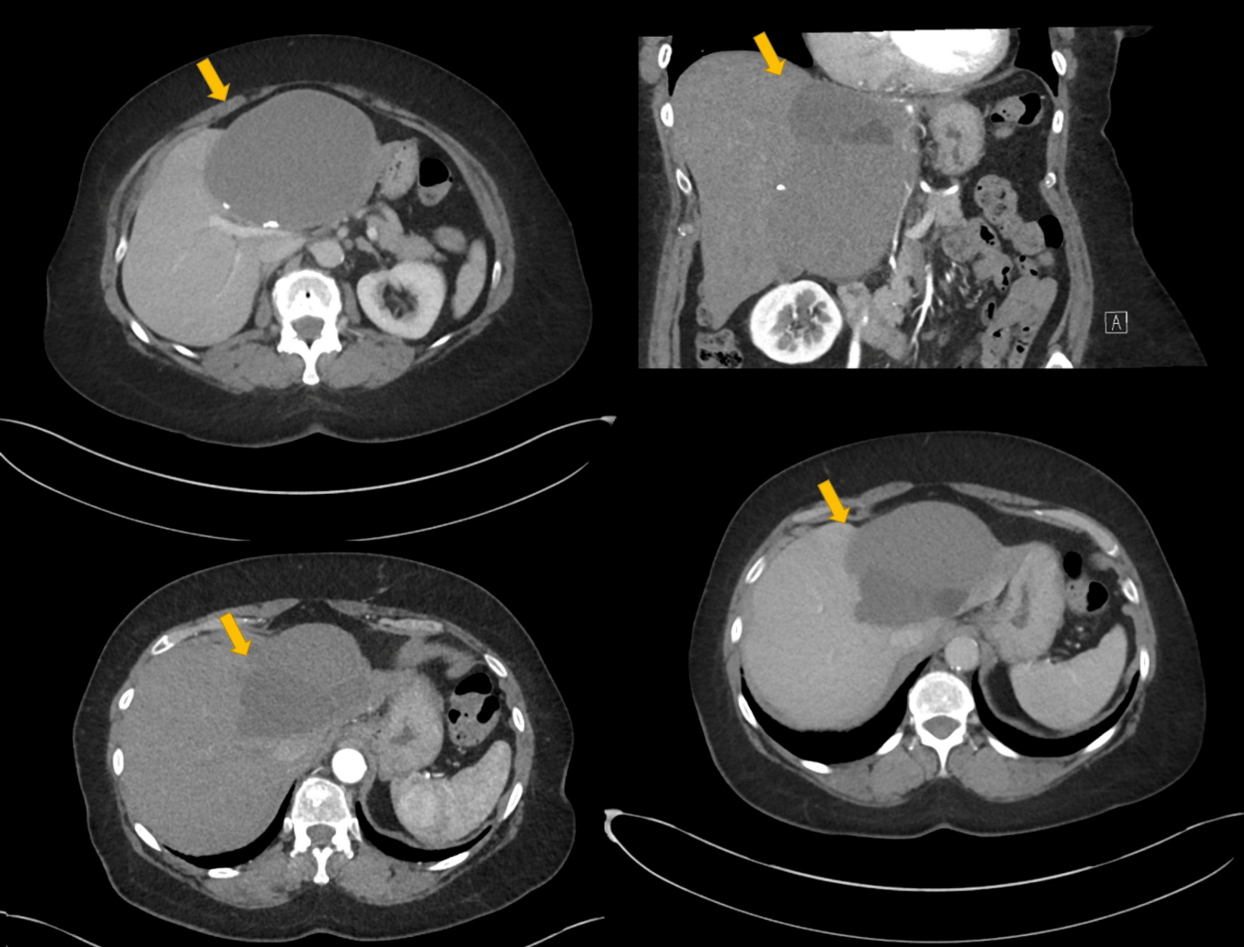
Axial and coronal CT images of BCAC. Axial (top left, bottom left, bottom right) and coronal (top right) contrast-enhanced CT images show a large, multiloculated cystic hepatic lesion (yellow arrows) with irregular wall thickening and subtle enhancing components. Internal septations and non-homogeneous fluid content are seen. These features were in keeping with malignant transformation, consistent with invasive BCAC. BCAC: biliary cystadenocarcinoma

**Figure 4:**
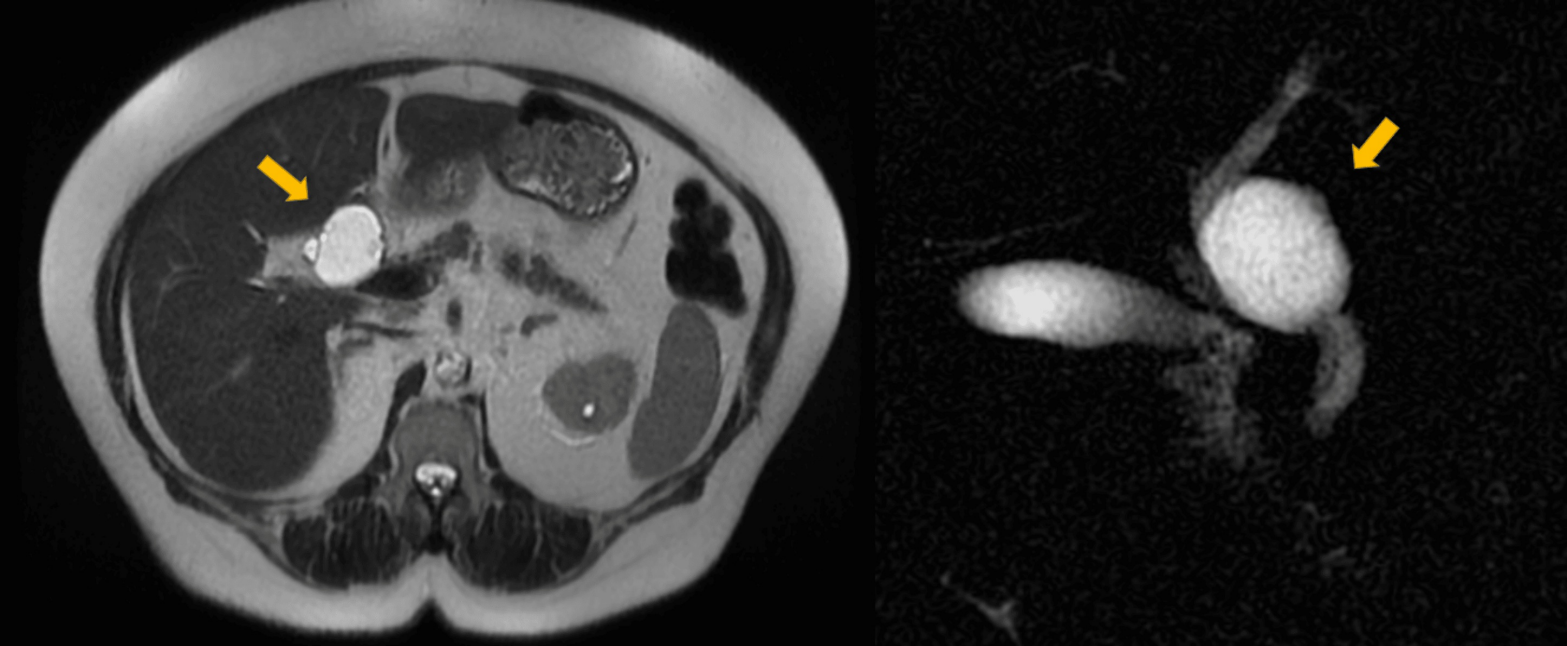
Extraheaptic BCA on MRI and MRCP. Axial T2-weighted MRI (left) and coronal MRCP (right) demonstrate a well-defined cystic lesion (arrows) arising from the extrahepatic bile duct, showing internal septations and direct communication with the biliary tree. The lesion was histologically confirmed as LGD BCA and represents the rare extrahepatic manifestation of biliary cystic neoplasms. MRCP: magnetic resonance cholangiopancreatography; LGD: low-grade dysplasia; BCA: biliary cystadenoma

Figures 1-4 demonstrate representative imaging features of BCAs in our cohort, including typical appearances of LGD BCA, HGD BCA, and invasive BCAC. Figure 4 specifically illustrates the rare extrahepatic BCAC case involving the common bile duct.

## Discussion

BCNs are rare cystic epithelial tumours of the liver and biliary tract, most commonly presenting as BCA and, less frequently, as BCAC [1]. Accounting for approximately 5% of intrahepatic cystic lesions, BCA predominantly affects middle-aged women, with a reported female-to-male ratio ranging from 4:1 to over 9:1, likely due to the defining presence of OTS in most cases [5,6]. Although historically considered benign, BCA carries malignant potential, with reported transformation rates to BCAC between 10% and 20%, particularly in longstanding or symptomatic lesions [6].

Differential diagnosis challenges

BCAs often present as large, multilocular cystic liver lesions, which can resemble a variety of benign and malignant hepatic entities, complicating radiological interpretation [7]. Lesion sizes typically range from 5 to 15 cm, contributing further to overlap with other pathologies [7,8]. The most frequent differential is a simple hepatic cyst, which is usually unilocular and thin-walled, although septations or haemorrhagic components may mimic the imaging appearance of BCA [6]. Hydatid cysts (echinococcosis), particularly in endemic regions, may also be confused with BCNs; while daughter cysts and calcifications are characteristic, atypical internal architecture can blur the distinction [9-12]. Another important mimic is intraductal papillary neoplasm of the bile duct (IPNB), which shares the multiloculated cystic morphology of BCA but typically shows biliary duct communication, a feature absent in classic BCAs [5]. Occasionally, cystic metastases from gastrointestinal or ovarian primaries may appear similar, especially if they lack obvious solid components or vascular invasion, raising diagnostic uncertainty [10]. Finally, degenerative changes in hepatocellular adenomas or hemorrhagic necrosis in other hepatic tumours can mimic a cystic neoplasm, further contributing to diagnostic difficulty [7]. Accurate differentiation is crucial, as management strategies diverge significantly between benign cysts, parasitic infections, and neoplasms with malignant potential [8].

Differentiation from simple cysts

Differentiating BCNs from simple hepatic cysts remains one of the most critical and common radiological challenges, given their overlapping appearances on cross-sectional imaging. Simple cysts are typically unilocular, thin-walled, and demonstrate homogeneous fluid content, with no enhancement on either CT or MRI. In contrast, BCNs more frequently display internal septations, capsular or septal enhancement, and variable internal fluid signal that often reflects mucinous or proteinaceous content [7]. Additional features such as mural nodules and coarse calcifications, though less commonly observed, raise concern for neoplastic pathology [6]. On MRI, BCN may exhibit T1 hyperintensity or heterogeneous T2 signal, often due to hemorrhagic or protein-rich cyst fluid, findings not typically seen in simple cysts [9]. When these imaging features are identified in combination, especially in symptomatic or enlarging lesions, surgical resection should be strongly considered due to the potential for malignant transformation [8]. Table 2 summarises radiological features of BCN as visualised on CT and MRI with representative differential diagnoses.

**Table 2:** Characteristic CT and MRI features of BCN and differential diagnoses. Radiological features of BCNs as visualised on CT and MRI, with representative differential diagnoses. Imaging findings are based on published literature and used in radiologic differentiation of simple cysts, neoplasms, and other cystic liver lesions. BCN: biliary cystic neoplasm; BCAC: biliary cystadenocarcinoma; IPNB: intraductal papillary neoplasm of the bile duct; HCC: hepatocellular carcinoma

Imaging feature	Typical appearance in BCNs	CT findings	MRI findings	Potential differential diagnoses
Wall morphology	Thin or thickened; enhancement may suggest a higher grade	Smooth or enhancing wall; irregularity may raise concern	T1 iso-/hypointense; enhancement post-contrast	Simple hepatic cyst, IPNB, cystic metastasis
Septations	Common, often thin; may enhance	Internal linear structures; may enhance if thick	T2 bright; may enhance on delayed images	Hydatid cyst (daughter cysts), multiloculated cysts
Mural nodules	Focal projections into the lumen; concern for malignancy	Soft-tissue nodule enhancing on contrast	T1 iso-/hyperintense; DWI variable	BCAC, cystic metastases, IPNB
Calcifications	May be coarse (capsular/septal); variable	High-attenuation foci; well-defined	Signal void on all sequences	Hydatid cyst, chronic abscess, degenerated cystic lesion
Internal fluid content	Mucinous or hemorrhagic; may be proteinaceous	High-density fluid (>20 HU) may suggest complexity	T1 hyperintense, T2 heterogeneous if hemorrhagic	Haemorrhagic cyst, cystic HCC, abscess, pseudocyst
Bile duct communication	Typically absent in classic BCN	Rarely visible	MRCP: no ductal continuity	IPNB (ductal communication), biliary hamartoma
Diffusion restriction	Not typical for benign BCA	Not applicable	Absent in most cases; may be present in high-grade/malignant	Abscess, cystic metastases

Comparison with literature findings

In our analysis, we grouped HGD and BCAC under the umbrella of “advanced histology,” based on their shared malignant potential and overlapping imaging characteristics. This classification aligns with current surgical pathology frameworks, which regard HGD as a precursor to invasive carcinoma. However, most prior radiologic series, including those by Mortelé and Wang, have traditionally compared benign BCA as a whole (without dysplasia subclassification) against overt carcinoma [6,7].

Radiological features typically associated with LGD BCA include well-defined, multilocular cystic morphology, thin septations, non-enhancing smooth walls, and the absence of mural nodules, features reported in over 80-90% of presumed benign cases [6]. By contrast, advanced histology (HGD or BCAC) is more frequently linked to irregular or thickened wall enhancement (up to 75%), mural nodules (30-50%), internal fluid heterogeneity or proteinaceous content (20-40%), and coarse septal or peripheral calcifications [7-9].

In our cohort of 42 patients, wall enhancement was observed in 67% of cases with advanced histology, compared to 28% in those with LGD. Complex (thick) fluid density was noted in 33% of advanced cases versus 8% in LGD. Interestingly, mural nodules were found exclusively in LGD (10%) and were absent in all advanced cases, an inverse pattern compared to prior reports [1]. Wall enhancement, while non-specific, may reflect stromal inflammation or early invasion and has been reported in both HGD and invasive lesions [11]. Septa enhancement, observed in 45% of our cohort, was distributed across all histologic categories and lacked discriminatory value, which is consistent with existing literature [8].

MRI signal characteristics such as T1 hypointensity and T2 hyperintensity were observed in 75% and 90% of cases, respectively, but were not predictive of histological grade. Diffusion restriction was absent in all lesions, and biliary duct communication was seen in 21%, without correlation to malignant transformation. These findings highlight a trend toward wall enhancement and internal fluid complexity as the most relevant imaging correlates of histologic advancement in BCN, while also underscoring the limitations of radiology in reliably excluding malignancy. All cases in our series were discussed within a dedicated HPB MDT, and resection was recommended for all complex or atypical cystic lesions.

Histology

In 10 of 42 cases (24%), OTS was not identified on histological examination of the resected specimens. According to the current WHO 2022 classification, the diagnosis of mucinous cystic neoplasm of the liver (MCN-L) requires the presence of mucin-producing epithelium and subepithelial OTS, a feature that distinguishes MCN from other cystic biliary neoplasms such as IPNB, which lack OTS and exhibit communication with the biliary tree [5,11].

Despite the absence of OTS, these 10 lesions were classified as BCA in the original pathology reports, based on their multilocular cystic architecture, mucinous epithelial lining, and lack of bile duct communication, features historically considered characteristic of BCAs before the adoption of stricter WHO criteria.

Several published series and pathological reviews have described similar lesions that fall outside the current WHO-defined categories, lacking both OTS and biliary communication [8,12]. These have been variously referred to as “biliary cystadenomas without ovarian-type stroma” or “cystic biliary neoplasms, not otherwise specified,” and are occasionally still diagnosed as BCA in routine practice, particularly when gross and microscopic morphology closely mirrors classical cases [2]. This diagnostic variability reflects both the evolving nature of hepatobiliary classification systems and the practical realities of real-world pathology reporting.

Significance of biliary cystadenocarcinomas

Although invasive BCAC is rare and understudied, available data suggest that affected patients generally experience poorer long-term outcomes compared to those with benign BCA, particularly in the absence of complete surgical resection [1,13]. In unresectable cases, palliative interventions such as biliary decompression or systemic therapy may be necessary to manage symptoms and slow disease progression [1,13]. When complete excision is achievable, favourable outcomes have been reported in small series, with one study noting five‑year survival rates approaching 100% and low recurrence following margin-negative resection [13,14].

Nonetheless, larger cohort studies indicate that recurrence remains possible and that BCAC carries a greater risk of adverse outcomes relative to BCA. Long-term survival appears to be influenced by both the completeness of resection and the underlying tumour biology [11]. Due to the rarity of BCAC, robust prognostic data are limited, and most survival figures derive from pooled case series or retrospective analyses. This underscores the need for larger, multi-institutional studies to better define morbidity, mortality, and long-term survival in invasive disease [11].

Limitations

This study is limited by its retrospective, single-centre design and relatively small sample size, which may restrict the generalisability of the findings. Histological subclassification was performed based on available pathology reports without re-review by expert gastrointestinal pathologists, which may introduce variability in grading. Additionally, radiological interpretation was not blinded or independently reviewed, potentially introducing observer bias. Finally, the absence of long-term follow-up data limits assessment of recurrence or progression, particularly in cases of advanced or borderline pathology.

## Conclusions

This study presents one of the largest single-centre series evaluating the radiologic-pathologic correlation in BCN, with a specific focus on identifying imaging features suggestive of histological grade. Our findings highlight that while features such as wall enhancement and complex internal fluid were more commonly associated with HGD or invasive BCAC, considerable overlap exists between benign and advanced lesions. In particular, mural nodules, commonly considered a worrisome sign in prior studies, were not predictive of advanced histology in our cohort, as all occurred in LGD BCA cases. Wall enhancement demonstrated the strongest diagnostic association with advanced disease, while thick fluid density, although infrequent, was highly specific. These results underline the limited reliability of any single imaging feature in isolation and reinforce the need for a comprehensive, multidisciplinary approach. Importantly, 24% of our histologically confirmed BCA cases lacked OTS, yet were still diagnosed as BCA based on architectural and cytologic features. This highlights the importance of a tailored, case-by-case diagnostic approach that considers the full radiologic-pathologic context, particularly when lesions do not meet all strict WHO criteria but exhibit typical features in clinical practice. Our data contribute to a growing body of literature suggesting that cystic biliary lesions may exist along a broader pathological spectrum than currently recognised. Given the malignant potential of BCN and the diagnostic uncertainty that persists even with advanced imaging, our study supports the continued recommendation for surgical resection of all complex or atypical cystic liver lesions. Future multicentre prospective studies and histopathological refinements are warranted to improve risk stratification and guide management more accurately in this rare but clinically significant group of liver tumours.
